# “Sneaky Spleen”: Three Cases of Ectopic Splenic Tissue Mimicking Neoplasia

**DOI:** 10.1155/crip/6644516

**Published:** 2025-09-15

**Authors:** Emily W. Gripp, Stacey M. Gargano

**Affiliations:** ^1^Sidney Kimmel Medical College at Thomas Jefferson University, Philadelphia, Pennsylvania, USA; ^2^Department of Pathology and Genomic Medicine, Thomas Jefferson University Hospital, Philadelphia, USA

**Keywords:** accessory spleen, case report, splenosis

## Abstract

Ectopic splenic tissue may arise as either a congenital anomaly or acquired seeding of fragments from a mechanically disrupted spleen. Regardless of the etiology, splenic tissue presenting at unexpected sites may lead to symptomatic or incidentally discovered lesions that may raise clinical suspicion for neoplasia. We present three cases of ectopic splenic tissue that were clinically ominous and necessitated pathologic tissue examination for definitive diagnosis.

## 1. Introduction

The spleen is an intraperitoneal organ normally situated in the left upper quadrant of the abdominal cavity [1]. Derived from the splanchnic mesoderm, the spleen begins to form during the fifth week of human embryonic development [2]. Its initial hematopoietic function ceases after the 14^th^ week of gestation, when the stage of lymphoid colonization begins and lays the foundation for its significant role in the immune system [1]. The size of a normal spleen in an adult can range from 7 to 14 cm in greatest dimension [2]. Microscopically, the spleen is comprised of two distinct compartments that serve various functions. In the red pulp, the splenic cords provide structural support, and the venous sinuses allow for effective filtration of the blood. The white pulp plays a role in the adaptive immune response and contains zones of B and T lymphocytes resembling lymph node architecture [3].

Additional foci of splenic tissue, derived from an error during development or fragmentation of the spleen with subsequent seeding and implantation, may be present in certain individuals, though the exact incidence is unknown and likely underestimated as most cases will remain asymptomatic and clinically undetected [4]. However, ectopic splenic tissue may sometimes produce symptoms or be discovered incidentally on radiographic imaging obtained for an unrelated workup [5, 6]. Here, we present three cases of ectopic splenic tissue masquerading as a more serious disease, which prompted tissue sampling for diagnostic evaluation.

## 2. Case Presentations

### 2.1. Case 1

A 62-year-old man with a history of splenectomy in his 20's was found to have an adrenal mass upon hematuria workup. MRI showed a 6.1 cm T2 hypointense left suprarenal mass concerning for neoplasm. The patient denied fevers, weight loss, palpitations, or headaches, and laboratory findings including a 24-h urine catecholamine test ruled out pheochromocytoma.

**Figure 1:**
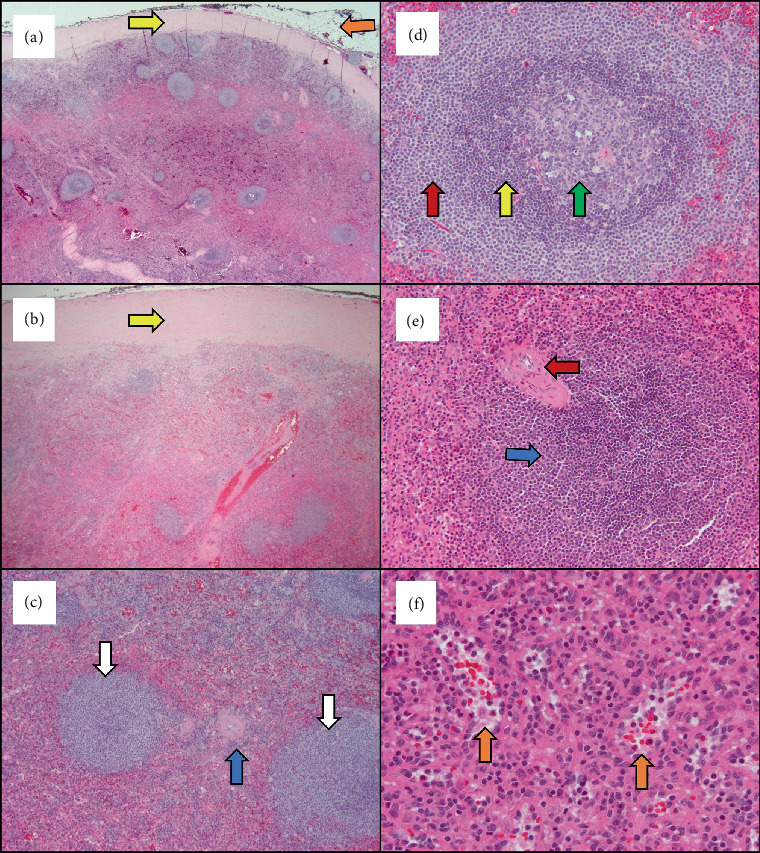
Case 1 microscopic findings, hematoxylin and eosin–stained slides. (a) Low magnification revealed a well-circumscribed mass with a thick fibrous capsule (yellow arrow) that was sharply demarcated from the adjacent adipose tissue (orange arrow) (2x). (b) Beneath the fibrous capsule (yellow arrow), the mass was composed of white pulp and red pulp characteristic of splenic tissue (4x). (c) Scattered lymphoid follicles (white arrow) and periarteriolar lymphatic sheath (blue arrow), comprising the white pulp compartment, were admixed with red pulp (10x). (d) Higher optical magnification of the white pulp showed an activated follicle composed of a germinal center (green arrow) surrounded by a dense rim of small lymphocytes representing the mantle zone (yellow arrow) and a more expanded rim of lymphocytes representing the marginal zone (red arrow) (20x). (e) Periarteriolar lymphatic sheath, consisting of mature lymphocytes (blue arrow) adjacent to arterioles (red arrow), was also identified (20x). (f) The red pulp was composed of a network of capillaries, venous sinuses (orange arrows) lined by cuboidal cells, and splenic cords (tissue between the sinuses) (40x).

Given the size of the mass and concern for possible malignancy, the patient underwent an exploratory laparotomy with excision of the mass. The pathology specimen consisted of a 7 cm encapsulated dark red mass with hemorrhagic cut surfaces, scant attached adipose tissue, and no apparent adrenal tissue. Histologic examination revealed that the mass was comprised of a thick fibrous capsule, splenic red pulp (capillaries, venous sinuses, and splenic cords) and white pulp (lymphoid follicles and periarteriolar lymphatic sheath [PALS]), consistent with a splenule (Figure 1).

Five years later, during workup for nephrolithiasis, the patient was noted to have enlarged gastrohepatic lymph nodes on CT scan. SPECT/CT of the abdomen was obtained following intravenous administration of technetium-99m (^99m^Tc) sulfur colloid. The soft tissue densities in the gastrohepatic space took up sulfur colloid, confirming they represented splenic tissue consistent with additional splenules.

### 2.2. Case 2

**Figure 2:**
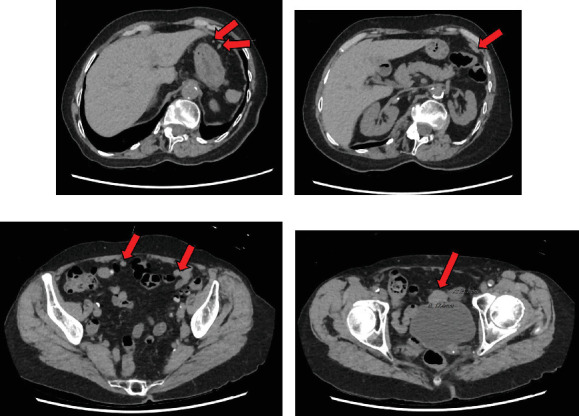
Case 2 radiographic findings. CT abdomen and pelvis (coronal view) showed multiple soft tissue lesions throughout the (a, b) omentum and (c, d) pelvic peritoneum, the largest located anterior to the bladder and measuring 2.5 cm.

A 72-year-old female patient with a history of traumatic splenectomy in childhood presented with 1 year of vague abdominal symptoms including lower abdominal cramping, nausea, and occasional vomiting. A CT scan of the abdomen and pelvis revealed multiple soft tissue densities throughout the omentum and peritoneum (Figure 2). The differential diagnosis included peritoneal carcinomatosis, but splenosis was another consideration, as the spleen was noted to be shrunken and irregular.

**Figure 3:**
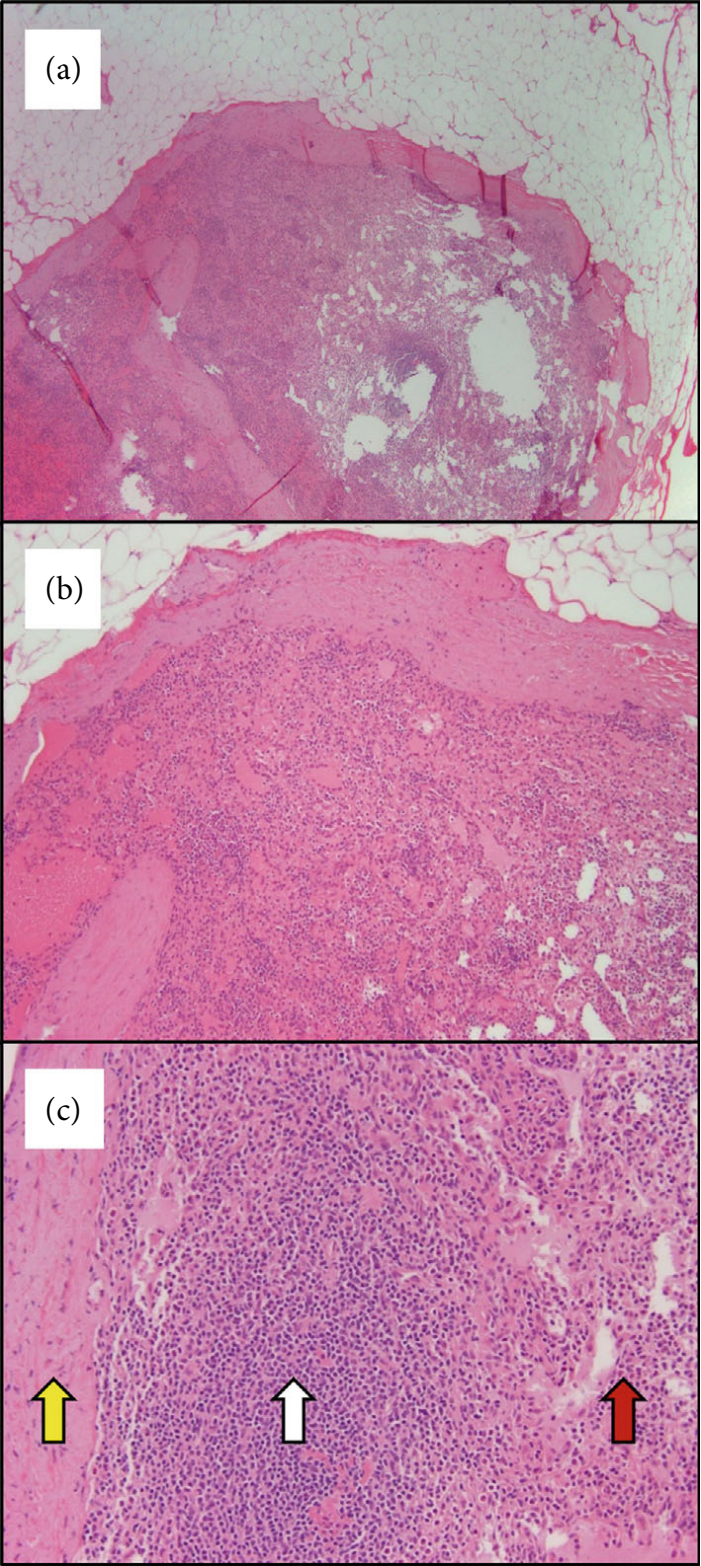
Case 2 microscopic findings, hematoxylin and eosin–stained slides. (a, b) Both peritoneal biopsies showed a well-circumscribed, encapsulated nodule within fibroadipose tissue (4x and 10x, respectively). (c) Higher optical magnification (20x) revealed that the nodules were composed of a fibrous capsule (yellow arrow), mature-appearing lymphocytes (white arrow), and splenic cords and sinuses (red arrow).

The patient underwent exploratory laparoscopy with biopsy of the two largest peritoneal lesions. Both biopsies showed adipose tissue containing splenic implants, consistent with abdominal splenosis (Figure 3).

### 2.3. Case 3

**Figure 4:**
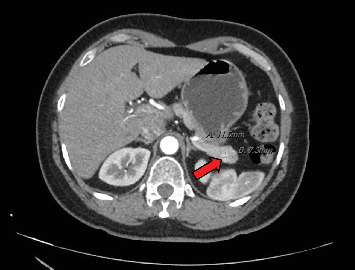
Case 3 radiographic findings. CT abdomen and pelvis with contrast showed a 1.2-cm arterial enhancing mass in the pancreatic tail (arrow). There was no pancreatic ductal dilatation, and the remainder of the pancreas showed normal enhancement.

A 56-year-old woman undergoing treatment for breast cancer had an incidentally discovered 1.2 cm arterial hyperenhancing mass in the tail of the pancreas on abdominal CT scan with contrast (Figure 4). The radiologic differential diagnosis included neuroendocrine tumor and metastasis. Subsequent octreotide scan showed increased activity in the region, suggestive of a somatostatin receptor-positive neuroendocrine tumor.

**Figure 5:**
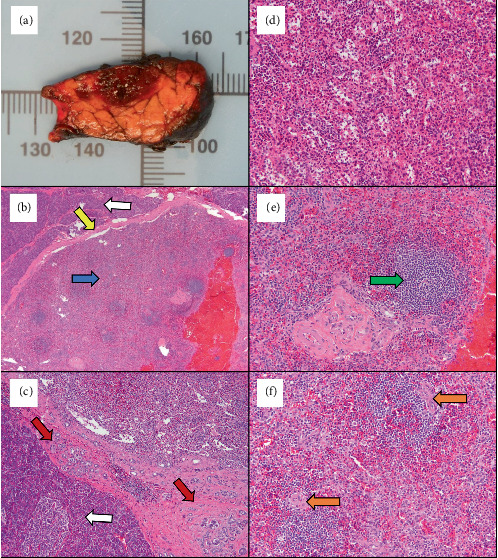
Case 3 gross and microscopic findings. (a) Cut section from the gross specimen showed a 1.5-cm well-circumscribed red-purple mass within the pancreatic parenchyma. (b) Histologic examination of hematoxylin and eosin–stained slides revealed splenic tissue (blue arrow) sharply demarcated from the pancreatic parenchyma (white arrow) by a fibrous capsule (yellow arrow) (4x). (c) Focally, the pancreatic parenchyma adjacent to the splenule showed fibrosis and loss of acinar tissue consistent with chronic pancreatitis (red arrows), while the remaining pancreatic tissue showed normal microanatomy (white arrow) (10x). The splenule consisted of (d) red pulp (20x), (e) scattered lymphoid follicles (green arrow) (20x), and (f) collections of mature lymphocytes adjacent to arterioles (orange arrows) consistent with periarteriolar lymphatic sheaths (20x).

The patient underwent distal pancreatectomy and splenectomy for resection of the mass. Pathologic evaluation was diagnostic of an intrapancreatic accessory spleen (Figure 5).

## 3. Discussion

In these three cases, patients underwent invasive procedures due to a clinical suspicion for possible neoplasia, yet microscopic examination confirmed the nature of the suspicious lesions to be ectopic splenic tissue. Two types of ectopic spleen are recognized: accessory spleen and splenosis.

An accessory spleen (also known as splenule or splenunculus) is an additional focus of splenic tissue in a separate location from the main organ, which is caused by incomplete fusion of mesenchymal buds during development. This phenomenon occurs in 10%–30% of people, and the most common locations are the hilum of the spleen (75%) and tail of the pancreas (20%) [4, 7]. Splenules are typically small and solitary, but they can be multiple, and compensatory hypertrophy may occur postsplenectomy (as seen in Case 1) [8]. The reported mean diameter of a splenule is highly variable, ranging from 1.0 to 3.5 cm [7].

Intrapancreatic accessory spleen is a known radiologic mimicker of pancreatic neuroendocrine tumor [9] (as demonstrated by Case 3) or metastatic lesion [10]. Interestingly, Rodriguez et al. [11] report a case of intrapancreatic accessory spleen that underwent fine needle aspiration (FNA) biopsy and demonstrated certain cytomorphologic features that further mimicked pancreatic neuroendocrine tumor. Other differential diagnoses that may be considered upon evaluation of an FNA sample from intrapancreatic accessory spleen include solid pseudopapillary neoplasm, lymphoepithelial cyst (if the sample includes only lymphocytes), lymphoproliferative disorder (if the lymphocytes show cytologic atypia), and hemangioma (if the specimen includes a predominant vascular component from sampling of the red pulp) [11, 12].

Splenosis is characterized by direct seeding of anatomic compartments (most commonly the peritoneal cavity) with splenic tissue derived from fragmentation of the spleen, secondary to traumatic rupture or splenectomy. Up to 65% of cases of splenic rupture may lead to abdominal or pelvic splenosis, with an average time interval of 10 years [13]. These lesions are generally multifocal, which, combined with their typical peritoneal localization, may simulate metastatic disease clinically (as shown in Case 2) [6, 13, 14]. Splenic implants have also been reported in unusual locations such as the kidney, liver, and even the brain, thus providing an opportunity to mimic a wide range of diagnostic entities [6].

In general, ectopic splenic tissue is an incidental finding that does not require surgical intervention, but it can be difficult to distinguish from neoplasia on imaging studies [4, 13]. Spleen-specific radiologic techniques, such as ^99m^Tc sulfur colloid scintigraphy, can be effective in identifying splenic tissue and thus avoiding biopsy [1, 8, 9]. However, certain cases will require tissue sampling to confirm the diagnosis and rule out a neoplastic process. In all three of our cases, the splenic tissue showed normal histology. More rarely, infarction or torsion of an accessory spleen may occur, leading to acute and severe abdominal pain and requiring surgical resection [5, 15, 16].

Grossly, spleens and splenules appear well circumscribed with a smooth fibrotic capsule and black–blue to dark red cut surfaces. Microscopically, splenic tissue appears as a mixture of red pulp and white pulp surrounded by a capsule of dense fibrous tissue. The red pulp consists of a network of capillaries, venous sinuses, and splenic cords, the latter of which contain macrophages, reticular cells, and plasma cells. The white pulp consists largely of lymphocytes, which are organized into a B-cell compartment (primary and secondary follicles) and T-cell compartment, known as the PALS. Other cell types found within the white pulp include macrophages, plasma cells, and dendritic cells [17]. Of note, any disease known to affect the spleen, including lymphoid hyperplasia, lymphoproliferative disorders, and various mesenchymal lesions, can also arise in accessory splenic tissue [8].

## 4. Conclusion

Awareness of the phenomenon of ectopic spleen is important for clinicians, radiologists, and pathologists. For the pathologist, attention to clinical history and recognition of normal splenic histology are key to the diagnosis.

## Data Availability

The data that support the findings of this study are available from the corresponding author upon reasonable request.
